# Effects of the GnRH agonist leuprorelin on liver volume in premenopausal women with severe polycystic liver disease

**DOI:** 10.1097/HC9.0000000000001067

**Published:** 2026-10-19

**Authors:** Inge G. Wijbenga, Renee Duijzer, Paul Geertsema, Sophie E. Aapkes, Thomas Bais, Lianne A. Messchendorp, Priya Vart, Robbert J. de Haas, Marjan van den Berg, Hans Blokzijl, Astrid E.P. Cantineau, Maatje D.A. van Gastel, Joost P.H Drenth, DrenthRon T. Gansevoort

**Affiliations:** 1 Division of Nephrology, Department of Internal Medicine, University Medical Center Groningen, University of Groningen, Groningen, The Netherlands; 2 Department of Gastroenterology and Hepatology, Radboudumc, Nijmegen, The Netherlands; 3 Department of Clinical Pharmacy and Pharmacology, University Medical Center Groningen, University of Groningen, Groningen, The Netherlands; 4 Department of Radiology, University Medical Center Groningen, University of Groningen, Groningen, The Netherlands; 5 Department of Obstetrics and Gynaecology, University Medical Center Groningen, University of Groningen, Groningen, The Netherlands; 6 Department of Gastroenterology and Hepatology, University Medical Center Groningen, University of Groningen, Groningen, The Netherlands; 7 Department of Gastroenterology and Hepatology, Amsterdam University Medical Center, Amsterdam, The Netherlands

**Keywords:** estrogens, GnRH agonist, leuprorelin, liver volume, PLD

## Abstract

**Background:**

Polycystic liver disease (PLD) is characterized by multiple liver cysts, sometimes leading to severe liver enlargement, with associated physical and psychosocial complaints. Treatment options are limited. Female gender is a risk factor for PLD; estrogen supplementation increases liver size in women with PLD, whereas the polycystic liver decreases after menopause. Estrogens may therefore be a target for therapy.

**Methods:**

In this prospective, open-label study, premenopausal women aged 18−45 years with severe PLD were randomized 1:1 to a direct start of the gonadotropin-releasing hormone agonist leuprorelin (n=6) or to standard care with delayed start of leuprorelin 18 months after randomization (n=8). The gonadotropin-releasing hormone agonist (11.25 mg injections once every 3 mo) leads to a reduction in estrogens and progesterone, and consequently to medically induced menopause. The primary outcome was liver growth rate at 18 months. Secondary outcomes comprised quality of life, menopausal side effects, and adverse events. An interim analysis was performed when all participants had reached month 18, as notable side effects were expressed.

**Results:**

In the group with direct start of leuprorelin, liver volume still increased in half of the participants, with the median liver growth rate not significantly different from the standard care group (4.04 [−4.62 to 6.84] vs. 11.0 [0.70−13.2] %/year, respectively, *p*=0.15). Importantly, PLD-related quality of life did not improve with treatment, whereas menopausal side effects were severe, leading one participant to stop treatment early. The total number of adverse events in the direct versus standard care group was 38 versus 9, respectively (*p*<0.001). Because of these results, the study was terminated early.

**Conclusions:**

leuprorelin treatment did not reverse liver volume, while menopausal side effects and adverse events were substantial.

## INTRODUCTION

Polycystic liver disease (PLD) is characterized by the progressive formation and growth of multiple liver cysts, leading to hepatomegaly.^1^ PLD can exist as an isolated disease in patients with autosomal dominant PLD or as an extrarenal manifestation in patients with autosomal dominant polycystic kidney disease (ADPKD). The prevalence of these subtypes causing PLD is approximately 1:158,000 and 1:2500,^1^,^2^,^3^,^4^,^5^,^6^ respectively. Of note, not all patients with ADPKD suffer from PLD.

In affected patients, liver volume increases progressively, although liver function generally remains intact.^7^ Related to the increased intra-abdominal volume, patients can suffer from serious physical and psychosocial complaints, such as reflux, decreased food intake, weight loss, sarcopenia, constipation, pain, and diaphragmatic, umbilical, and inguinal herniations.^1^,^8^,^9^,^10^ In addition, there are related liver-specific complications such as cyst bleeding, rupture, and infections. Furthermore, a large abdominal volume can cause a distorted body image and related psychological complaints.^1^,^7^ Together, these signs and symptoms result in a decreased quality of life, which can be serious depending on the volume of the enlarged liver.^11^,^12^

Currently, there are several surgical treatment options available to reduce liver volume and relieve symptoms in case of predominant liver cysts, such as cyst sclerotherapy and/or fenestration, and hemihepatectomy. To date, the only drug treatment available with proven efficacy to halt or even reverse the growth of liver cysts is somatostatin analogs, such as octreotide and lanreotide.^13^ Nevertheless, in ~40% of patients with PLD, growth in liver volume continues despite the use of these drugs.^8^ Moreover, not all patients tolerate somatostatin analog treatment because of drug-related side effects, such as injection site–related pain, bradycardia, nausea, abdominal cramps, diarrhea, cholelithiasis, and pancreatitis.^13^,^14^ The last resort option in patients with large livers and ongoing liver growth is liver transplantation, with associated morbidity and mortality. Therefore, there is a need for new treatments to halt liver growth in PLD.

Epidemiological and experimental evidence point to a pathophysiological role of estrogens in the development of liver cysts in PLD. Female gender is the most important risk factor for PLD, with larger liver volumes in women than in men, and more women requiring a liver transplantation.^15^,^16^,^17^,^18^,^19^ Estrogen supplementation has been shown to lead to enhanced liver growth,^20^ and when estrogen levels fall after menopause, liver volumes sometimes decrease.^21^ In vitro evidence indicates that hepatic cyst epithelium expresses functional estrogen receptors, with estrogen stimulation promoting, and estrogen blockade inhibiting cystic epithelial cell proliferation in ADPKD-derived cultures.^7^,^18^,^22^,^23^,^24^ This body of evidence has led to the hypothesis that estrogens are involved in the pathophysiology of PLD and may be a target for therapy.^7^

To test this hypothesis, we designed the AGAINST-PLD (acronym for A GnRH Agonist IN premenopausal women Study to Treat Polycystic Liver Disease) trial in which young women with severe PLD were treated with the GnRH agonist leuprorelin. This drug downregulates the GnRH receptor in the pituitary gland, which ultimately leads to a reduction of estrogen and progesterone levels, aiming to reverse liver growth, with medically induced menopause as an inherent consequence.

## METHODS

### Study design and participants

The AGAINST-PLD study is an investigator-driven, phase IIb, prospective, randomized, open-label clinical trial with blinded endpoint analysis, which was performed in 3 Dutch University Medical Centers (UMC Groningen, Radboudumc, and Amsterdam UMC). Details of the study protocol have been published previously.^25^ The trial was conducted in accordance with the International Conference of Harmonization of Good Clinical Practice Guidelines and the Declarations of Helsinki and Istanbul. Ethical approval was obtained in all participating centers, with the institutional review board of the UMC Groningen acting as the central institutional review board (METC 2021/119). All participants gave written informed consent before any study-related procedures were performed.

Patients were screened for inclusion and exclusion criteria. The main inclusion criteria were premenopausal women, aged 18−45 years, with a very large liver for age (defined as the upper 10% of PLD liver volumes in specific age categories in our observational registry^26^) or age-independent height-adjusted liver volume (hTLV) >2.0 L/m and Polycystic Liver Disease Questionnaire (PLD-Q) score >30. Anti-Müllerian hormone level had to be >0.3 ng/mL to ensure the premenopausal state. Patients could be included if on stable treatment with a somatostatin analog (without initiation or discontinuation during the study), if they had a contraindication, intolerance, or proven inefficacy to this drug, or if they refused treatment. Exclusion criteria were, among others, active desire to have children during the study, any symptoms of (upcoming) menopause, use of exogenous estrogen and/or progesterone during the 6 months before screening (including oral anticonceptives and intrauterine devices), kidney transplantation, and liver transplantation or liver surgery expected within 1.5 years.

The design of the study was determined after discussion with 2 focus groups of young women with severe PLD, led by an independent social scientist with expertise in carrying out such focus group meetings.

### Interventions

The study was designed as a 3-year study, with participants to be randomized 1:1 to either direct start with the GnRH agonist leuprorelin treatment given as a subcutaneous injection at a dose of 11.25 mg every 3 months or standard care with delayed start, meaning starting leuprorelin 18 months after randomization (Figure 1). The injections were administered by the participants themselves at home or by study personnel, depending on the preferences of the participants. Leuprorelin was started during the luteal phase of the menstrual cycle to avoid a paradoxical surge in estrogen and progesterone that can be seen after the first administration. Randomization to the 2 treatment groups was conducted using an online interactive response system tool, stratified by somatostatin analog use (yes/no) and age (<40 and ≥40 y). Blood pressure treatment was targeted to below 130/80 mm Hg with Renin-Angiotensin-Aldosteron System (RAAS) inhibitors as the first-choice agents. To prevent osteoporosis, calcium and vitamin D (500 mg/800 IE) were prescribed to all participants.

**FIGURE 1:**
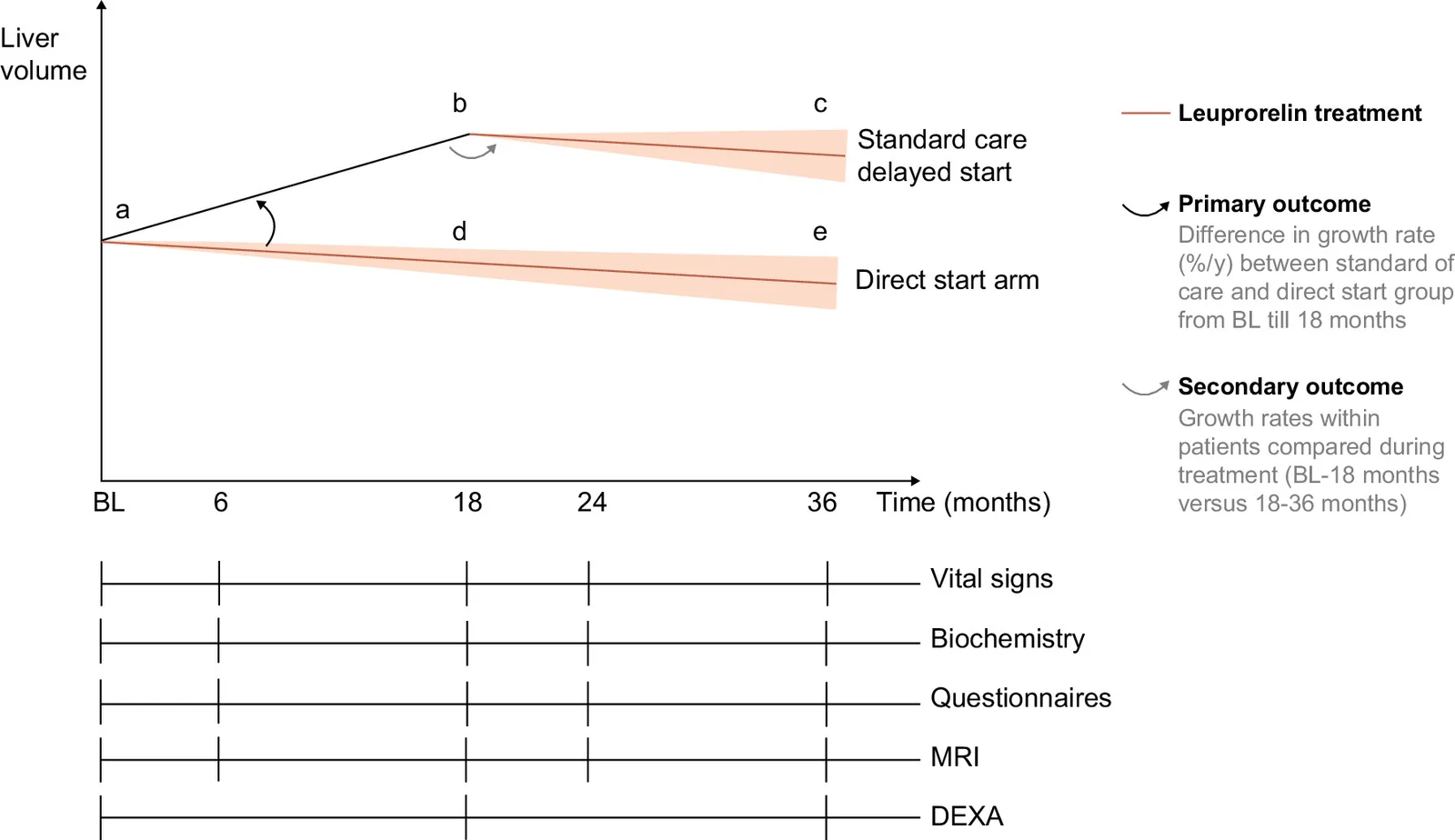
Study design of the AGAINST-PLD study. Patients are randomized between direct and standard care (after 18 months) of treatment. a=BL scan. Primary outcome: comparison between growth on treatment and without treatment in the first 18 months is a, b versus a, d. Abbreviations: AGAINST-PLD, A GnRH Agonist IN premenopausal women Study to Treat Polycystic Liver Disease; BL, baseline​​​​​​; DEXA, Dual-Energy X-ray Absorptiometry.

### Procedures

Data were collected during study visits at the participating medical centers at baseline, month 6, and month 18. These data included demographic information, medication use, laboratory findings, and imaging data. All plasma values were obtained using standard laboratory methods. Total liver volume (TLV) and total kidney volume were determined by an abdominal MRI. TLV and total kidney volume were assessed using an automated MRI program by trained reviewers blinded to patient identity, treatment allocation, and order of study visit.^27^ Measured volumes are adjusted for height. A more detailed description of the methodology, accuracy, and precision of the TLV and total kidney volume measurement has been published previously.^25^,^27^ Supplemental Figure S1, https://links.lww.com/HC9/C504, shows a representative MR image to facilitate the seriousness of the disease and the methodology of volume measurement. At the same time points, bone densitometry was performed by a dual-energy x-ray absorptiometry scan, and quality of life was determined using questionnaires. The collected data were managed using Research Electronic Data CAPture (REDCap) software.

### Outcomes

The primary outcome was liver growth (%/year) calculated over the first 18 months of the trial and compared between the direct-start group (on treatment) and standard-of-care group (a–b vs. a–d, Figure 1 ^25^). Secondary outcomes were changes in PLD disease severity, as assessed using validated instruments, namely the PLD-Q^12^ (with a score between 0 and 100; higher scores indicate greater PLD-related complaints and severity). Quality of life and symptoms of depression were assessed by the 36-Item Short Form Health Survey (SF-36, with a score between 0 and 100; higher scores indicate a better quality of life) and the Beck Depression Inventory II questionnaire (with a score between 0 and 63; higher scores indicate more severe depressive symptoms). Potential menopausal side effects of leuprorelin treatment were measured by the Menopause Specific Quality of Life (MENQOL) questionnaire (with a score between 0 and 6; higher scores indicate more menopause-related complaints). Also, the tolerability of the study medication and incidence of (serious) adverse events were monitored. Exploratory outcomes were differences in hTLV at various time points in the study and changes in bone density (assessed using the T-score on dual-energy x-ray absorptiometry scan) between the 2 treatment groups. In addition, kidney growth rates and volumes were assessed in participants with ADPKD. Because of the anticipated impact of study treatment, it was decided a priori that an interim analysis would be performed when half of the women to be included (n=18) had completed half of the study (ie, passed the month 18 time point).

### Statistical analysis

A sample size calculation was performed using data from our observational cohort study, which showed a mean liver growth rate of 5.8±2.8% per 18 months among patients meeting the inclusion criteria.^8^ Since the information on liver growth rate in this specific patient group is scarce, we conservatively doubled the SD to ensure enough power. Aiming to at least stop liver growth, using a β of 80% and an α of 0.05, and incorporating a 20% drop-out rate, it was calculated that inclusion of 36 patients would be needed.^25^ In other proof-of-concept studies in PLD, similar numbers of patients were included.^25^,^28^,^29^

Baseline characteristics are provided for categorical variables as numbers (and percentages) and for continuous variables as median (interquartile interval). The primary analysis of change in liver and kidney volume was performed using the Wilcoxon signed-rank test. To test the robustness of our primary analysis, we performed an additional analysis using linear mixed models. An interaction term of time (in years) with treatment group and baseline height-adjusted TLV, baseline age, somatostatin use, and autosomal dominant PLD/ADPKD status were the fixed effects in these models. Time (in years) and participants were random effects. The intercept and slope were allowed to vary randomly with an unstructured covariance matrix. Ninety-five percent CIs and *p* values were used for estimates. Due to the limited number of participants in the interim analysis, these analyses are performed as a per-protocol analysis instead of an intention-to-treat analysis. Nominal data were compared using the Mann-Whitney *U* test. Analysis of the incidence of adverse events was done as the total number of adverse events, per category, as well as per group. A comparison was made between the direct start of treatment and the standard of care. Statistical significance was defined as a 2-sided *p* value of <0.05. All statistical analyses were performed using R version 4.4.2 and SPSS version 28.0.1.0.

## RESULTS

### Study population

Figure 2 shows the flow diagram of the study. Sixteen women were screened, of whom 2 did not meet the study’s inclusion and exclusion criteria. Fourteen women were randomized, of whom 6 were assigned to the direct start of leuprorelin treatment and 8 to standard of care. In the direct-start group, 1 participant discontinued medication after 8 months due to severe menopausal symptoms. However, study visits were continued. In the standard-of-care group, 1 participant discontinued the study after 12 months because she received a liver transplantation. During the study, a high number of participating women experienced adverse events in the direct-start group. Therefore, the a priori determined interim analysis was performed earlier, when 14 rather than 18 participants completed half of the study. The per-protocol analyses were conducted with 8 participants in the standard-of-care group and 6 participants in the direct-start group for the first study period (0–6 mo) and 7 participants in the standard-of-care group and 5 participants in the direct-start group for the second study period (6–18 mo). Based on the results of this interim analysis, the independent Data Safety Monitoring Board advised terminating the study early, to which the Steering Committee and institutional review board agreed. Hence, this interim analysis is the basis for the present study report. After this decision, and being informed about the results obtained, only 2 out of 14 participating women decided to receive leuprorelin treatment in the framework of clinical care, both with a serial MRI-proven decrease in liver volume.

**FIGURE 2:**
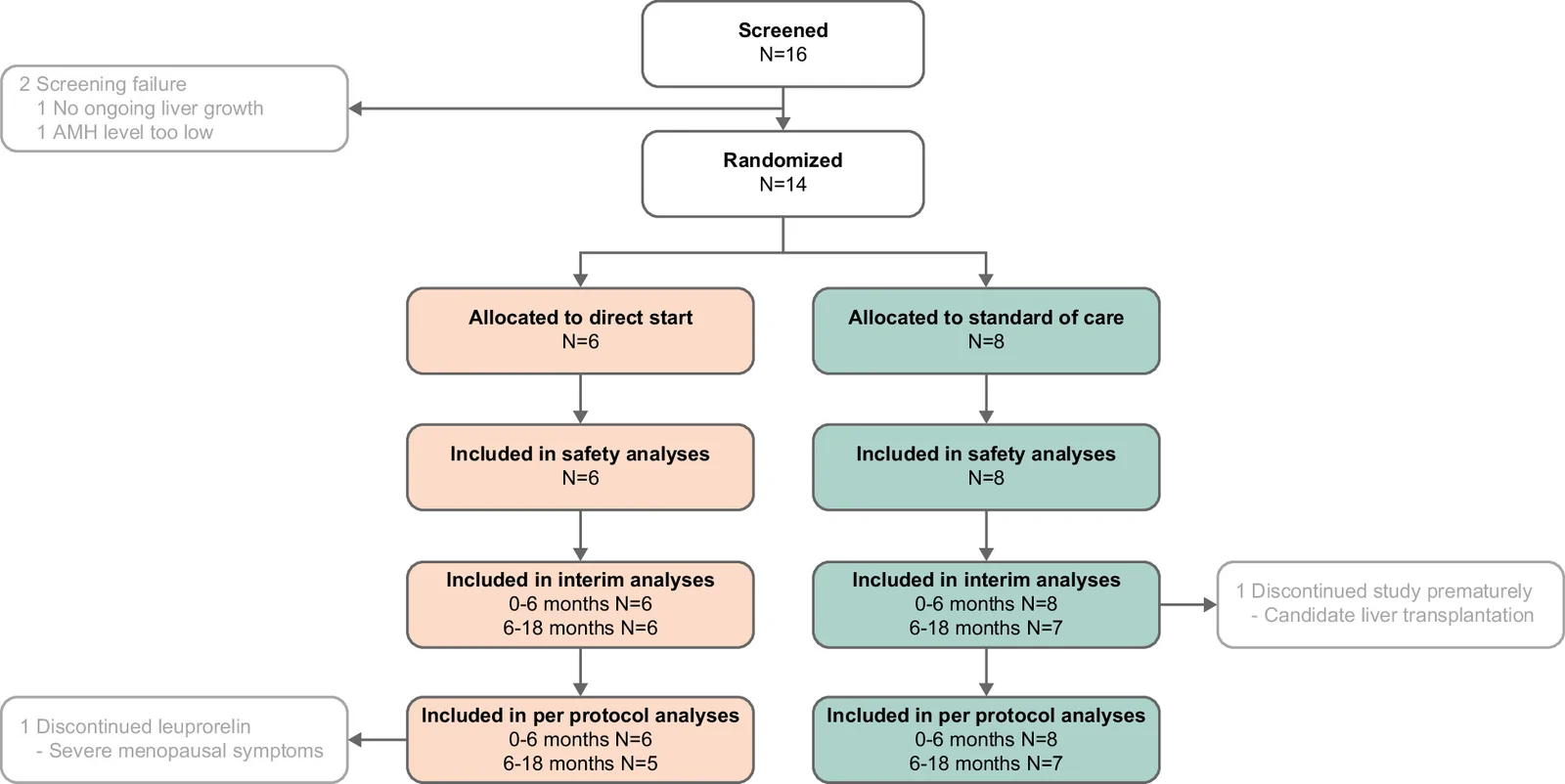
Study enrollment and outcomes. Abbreviation: AMH, anti-Müllerian hormone.

### Characteristics of the study population

The baseline characteristics of the study population are shown in Table 1. There were no differences in age (median 43 [interquartile interval: 38–45] vs. 41 [39−41] y) and body mass index (24.5 [22.9−28.0] vs. 23.7 [20.8−24.6] kg/m^2^) between the standard-of-care and direct-start group, respectively. A slightly larger hTLV was seen in the participants of the standard-of-care group compared with the direct-start group, which was not statistically significant (3.87 [3.18−4.46] vs. 2.90 [2.49−3.93] L/m, *p*=0.23).

**TABLE 1:** Baseline characteristics according to treatment group

	Leuprorelin	
	Standard of care (N=8)	Direct start (N=6)
Caucasian, n (%)	6 (75.0)	5 (83.3)
Age (y)	43 (38−45)	41 (39−41)
BMI (kg/m^2^)	24.5 (22.9−28.0)	23.7 (20.8−24.6)
Upper arm circumference (cm)	25.0 (22.8−27.8)	25.0 (23.3−26.5)
Diagnosis		
ADPLD	1 (12.5)	2 (33.3)
ADPKD	7 (87.5)	4 (66.7)
PLD-related outcomes		
Liver volume (L/m)	3.87 (3.18−4.46)	2.90 (2.49−3.93)
History of liver pain, n (%)	7 (87.5)	4 (66.7)
History of liver cyst infection, n (%)	0	0
History of liver cyst bleeding, n (%)	1 (12.5)	0
Liver volume–influencing factors		
Estrogen and/or progesterone use in the past, n (%)		
Oral contraception	8 (100)	6 (100)
Hormone-containing IUD	5 (62.5)	2 (33.3)
Unilateral ovariectomy, n (%)	0	0
Pregnancies in the past	8 (100)	4 (66.7)
Number of pregnancies	3 [2.8−3.0]	2 [1.8−2.0]
Hemihepatectomy, n (%)	0	0
Aspiration and/or sclerotherapy liver cyst(s), n (%)	0	2 (33.3)
Use of somatostatin analogs, n (%)		
Current	2 (25)	0
Past	2 (25)	1 (16.7)
Never	4 (50)	5 (83.3)
Biochemistry		
Anti-Müllerian hormone (μg/L)	0.90 (0.37−1.80)	2.00 (0.67−2.76)
LH (U/L)	6.8 (4.8−8.2)	39.3 (32.1−55.5)
Estradiol (nmol/L)	0.28 (0.23−0.32)	0.49 (0.39−0.63)
Progesterone (nmol/L)	8.60 (0.18−26.0)	43.5 (15.4−55.1)
ASAT (U/L)	22 (20−29)	32 (21−46)
ALAT (U/L)	19 (12−25)	28 (23−59)
AF (U/L)	84 (67−108)	77 (55−181)
GGT (U/L)	75 (46−119)	75 (37−413)
Total bilirubin (μmol/L)	14 (11−23)	15 (9−20)
CA 19.9 (kU/L)	16 (10−27)	12 (2−33)
PKD-related characteristics^a^		
Total kidney volume (mL/m)	456 [354−555]	719 [465−1079]
eGFR (mL/min/1.73 m^2^)	73.1 (65.0−111)	92.2 (77.3−113)
Genetic variant causing PLD, n (%)		
PKD-1	2 (25.0)	0
SEC. 63	0	1 (16.7)
Unknown	6 (75.0)	5 (83.3)

*Note:* Variables are presented as number (%) or as median and IQI.

^a^ Only including subjects with ADPKD (7 vs. 4).

Abbreviations: ADPKD, autosomal dominant polycystic kidney disease; ADPLD, autosomal dominant polycystic liver disease; AF, alkaline phosphotase; BMI, body mass index; CA 19.9, carbohydrate antigen 19-9; eGFR, estimated glomerular filtration rate; IQI, interquartile interval; IUD, intrauterine device; LH, luteinizing hormone; PKD, polycystic kidney disease; PLD, polycystic liver disease.

### Liver growth

Average liver growth rate in the direct-start group was somewhat lower, but still positive and not significantly different from the standard-of-care group across all study periods, with the primary outcome of change in TLV at baseline versus month 18 being 4.04 (−4.62 to 6.84) versus 11.0 (0.70−13.2) %/year, respectively (difference *p*=0.15) (Figure 3A and Table 2). In the direct-start group, 3 out of 6 participants still showed an increase in TLV with treatment. Essentially similar results were obtained when comparing liver growth rates using linear mixed-effects models as a sensitivity analysis. Also, the intention-to-treat analysis and the analysis of liver growth using Student *t* test showed similar results (Supplemental Tables S4 and S1, respectively, https://links.lww.com/HC9/C504).

**FIGURE 3:**
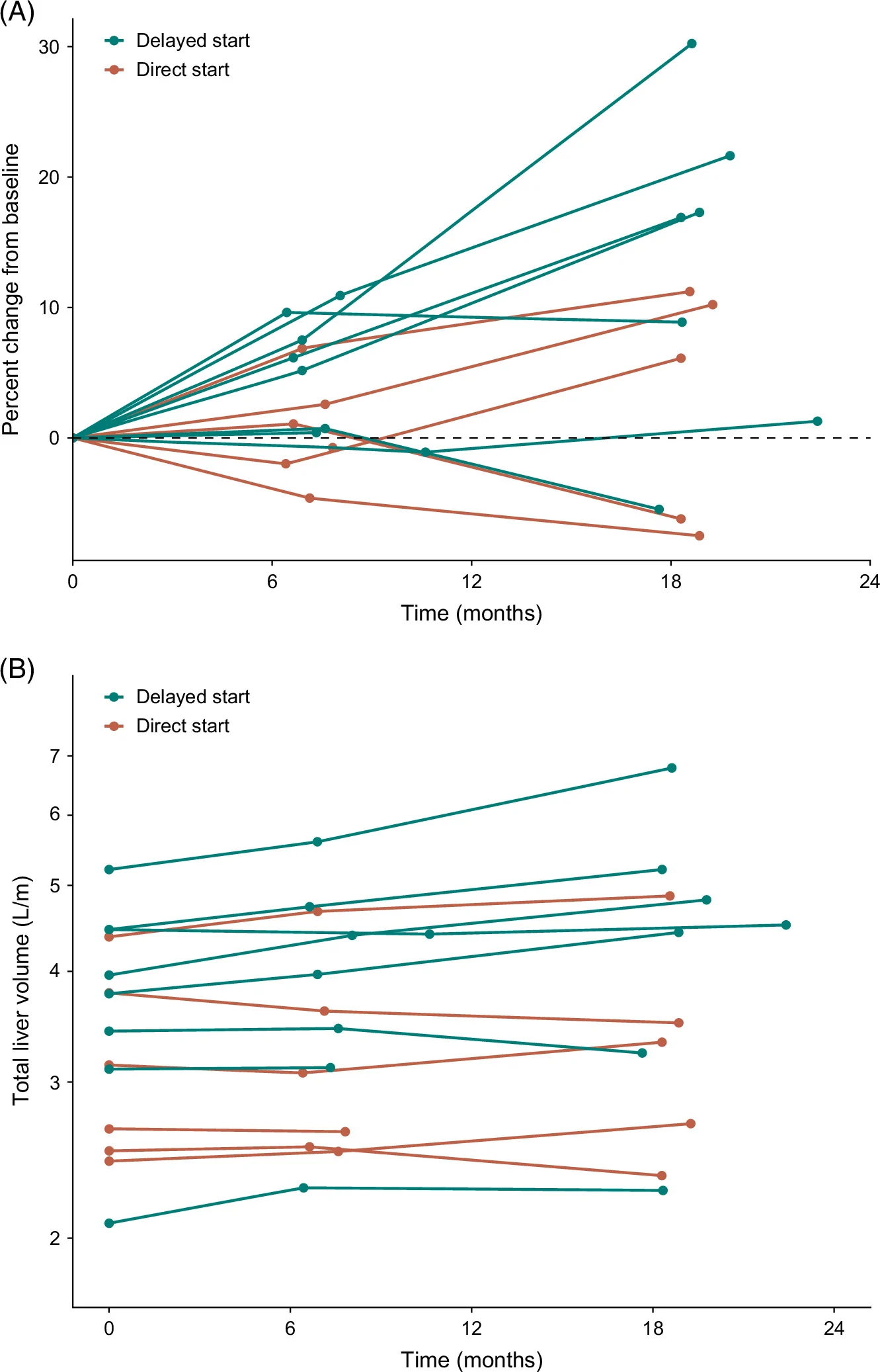
Height-adjusted total liver volume over time in premenopausal female patients with severe polycystic liver disease with direct start (0−6 mo: n=6, 6−18 mo: n=5) or standard of care (0−6 mo: n=8, 6−18 mo: n=7 ) of leuprorelin treatment. (A) Percent change from baseline. (B) Change in absolute height-adjusted total liver volume.

**TABLE 2:** Liver volumes according to treatment group

	Leuprorelin				
	Standard of care		Direct start		
	A		A		*p*
Primary outcome					
Liver growth rate (%/year)					
First study period (0−6 mo)	8	11.6 (0.83–16.5)	6	0.45 (−4.88 to 6.90)	0.14
Second study period (6−18 mo)	7	10.6 (−0.74 to −12.6)	5	4.46^a^ (−5.57 to 8.60)	0.27
Total study period (0−18 mo)	7	11.0 (0.70−13.2)	5	4.04 (−4.62 to 6.84)	0.15
Linear mixed effect models (%/year, CI)					
First study period (0−6 mo)	8	8.02 (2.49–14.1)	6	0.93 (−5.27 to 7.78)	0.15
Second study period (6−18 mo)	7	6.55^b^ (1.36–12.1)	5	1.64^b^ (−4.14 to 7.80)	0.26
Total study period (0−18 mo)	8^c^	7.04 (2.36–12.0)	6^c^	1.21 (−3.96 to 6.57)	0.14
Exploratory outcomes					
Height-adjusted total liver volume (L/m)					
Baseline	8	3.87 (3.18−4.46)	6	2.90 (2.49−3.93)	0.23
6 mo	8	4.17 (3.20−4.65)	6	2.86 (2.53−3.87)	0.18
18 mo	7	4.51 (3.24−5.21)	5	3.33 (2.52−4.18)	0.34

*Note:* Variables are presented as median (IQI, interquartile interval). *p* values are calculated using the Mann-Whitney *U* for cross-sectional comparisons. For paired analyses, the Wilcoxon signed-rank test was used. Data of all patients were complete.

^a^ First versus second study period, *p*=0.50 (standard of care) and *p*=0.89 (direct start).

^b^ First versus second study period, *p*=0.64 (standard of care) and *p*=0.85 (direct start).

^c^ Liver growth rate during the total study period, as estimated by linear mixed-effects modeling, included additional measurements of the first study period from 1 patient in the standard-of-care group and 1 patient from the direct-start group.

Abbreviation: A, available.

### Quality of life and treatment side effect questionnaires

At baseline, 6 months, and 18 months, the quality of life and treatment side effect questionnaires (PLD-Q, SF-36, Beck Depression Inventory II, and MENQOL) were collected (Tables 3 and 4). There was no difference in PLD-Q score or changes in the score between the direct-start group and the standard-of-care group at any time point. Similarly, the SF-36 physical and mental component scores did not differ between groups, nor did the Beck Depression Inventory II score. For menopausal complaints, as assessed with the MENQOL questionnaire, there were no differences at baseline in overall scores or in any domain-specific scores between the standard-of-care group and the direct-start group. At 6 months, the vasomotoric symptoms and sexual health scores were significantly worse in the direct-start group (3.33 [0.92−5.25] and 1.83 [0.92−5.50]) compared with the standard-of-care group (0.00 [0.00–0.00], *p*=0.002 and 0.33 [0.00–0.75], *p*=0.009). This was also the case at 18 months. When looking at trends over time, the score in sexual health in the direct-start group worsened over 18 months (0.83 [0.08−3.33], *p*<0.01), whereas a trend toward improvement was seen in the standard-of-care group (−0.70 [−1.80 to 0.00], *p*=0.05). No significant difference was seen in absolute values or in the change of menopause-related psychological and other physical symptoms between the 2 groups (Table 4).

**TABLE 3:** Results of quality of life questionnaires according to treatment group

	Leuprorelin						
	Standard of care			Direct start			
	A	NA		A	NA		*p*
PLD-Q score							
Baseline	7	1	51.7 (46.5−70.7)	6	—	52.5 (42.8−64.2)	0.73
6 mo	7	1	54.0 (46.0−57.6)	5	1	50.5 (46.0−71.0)	0.88
18 mo	7	1	52.0 (48.0−55.1)	6	—	47.2 (38.8−58.2)	0.45
Change (6 mo to baseline)	6	2	−2.0 (−10.9 to 4.3)	5	1	2.0 (−6.4 to 10.9)	0.54
Change (18 mo to baseline)	6	2	−1.6 (−10.7 to 4.8)	6	—	−5.1 (−12.1 to 1.1)	0.59
SF-36 score							
Baseline	6	2		5	1		
PCS			43.3 (36.3−45.6)			44.7 (34.1−45.6)	0.79
MCS			47.3 (34.3−50.9)			46.3 (28.5−50.3)	0.93
6 mo	6	2		6	—		
PCS			40.2 (37.2−43.9)			43.3 (36.2−48.7)	0.49
MCS			48.0 (36.3−52.2)			46.4 (31.8−51.1)	0.94
18 mo	7	1		6	—		
PCS			41.8 (33.7−46.9)			47.5 (37.9−50.2)	0.45
MCS			47.5 (43.7−51.3)			49.0 (40.3−53.2)	0.73
Change (6 mo to baseline)	4	4		5	1		
PCS			−5.8 (−10.9 to −3.6)			−1.9 (−4.9 to 1.5)	0.11
MCS			2.6 (−4.4 to 9.1)			−2.3 (−5.6 to 4.5)	0.73
Change (18 mo to baseline)	5	3		5	1		
PCS			−3.1 (−9.4 to 7.6)			0.7 (−1.1 to 4.1)	0.55
MCS			−0.06 (−7.0 to 8.3)			3.2 (−4.8 to 15.4)	0.69

*Note*: Variables are given as median and interquartile interval. *p* values were calculated using the Mann-Whitney *U* test. Numbers of patients differ from those in Table 2 because for some patients, data on quality of life were missing (for the delayed and direct start of treatment at baseline, n=6−7 and n=6−5; at month 6, n=6−7 and n=5−6; and at month 18, n=7 and n=6, respectively).

Abbreviations: A, available MCS, mental component summary; NA, not available; PCS, physical component summary; PLD-Q, Polycystic Liver Disease Questionnaire; SF-36, Short Form Health Survey.

**TABLE 4:** Results of side effect questionnaires according to treatment group

	Leuprorelin						
	Standard of care			Direct start			
	A	NA		A	NA		*p*
BD-II score							
Baseline	5	3	11.0 (4.5−17.0)	4	2	7.5 (3.3−12.5)	0.56
6 mo	6	2	6.5 (3.8−10.3)	6	—	9.5 (4.3−17.5)	0.49
18 mo	7	1	9.0 (4.011.0)	6	0	5.5 (2.5−19.8)	0.63
Change (6 mo to baseline)	3	5	0.0 (-11.0 to 5.0)	4	2	0.0 (−1.8 to 5.5)	0.86
Change (18 mo to baseline)	4	4	2.0 (0.0−5.5)	4	2	0.5 (−7.8 to 2.8)	0.34
MENQOL score							
Baseline	5	3		4	2		
Overall score			1.38 (0.41−1.88)			0.74 (0.59−0.92)	0.73
Vasomotor symptoms			0.00 (0.00–0.33)			0.50 (0.00−1.00)	0.41
Psychological symptoms			1.57 (0.14−2.36)			1.14 (0.29−1.46)	0.56
Physical symptoms			1.44 (0.69−2.06)			0.72 (0.59−0.98)	0.19
Sexual health			0.00 (0.00−2.33)			0.17 (0.00−1.08)	0.91
6 mo	6	2		6	—		
Overall score			0.67 (0.43−1.10)			1.79 (0.77−3.49)	0.04
Vasomotor symptoms			0.00 (0.00−0.00)			3.33 (0.92−5.25)	0.002
Psychological symptoms			0.36 (0.11−1.04)			1.36 (0.36−3.54)	0.09
Physical symptoms			1.06 (0.63−1.45)			1.59 (0.97−2.83)	0.24
Sexual health			0.33 (0.00−0.75)			1.83 (0.92−5.50)	0.009
18 mo	7	1		6	—		
Overall score			0.86 (0.38−1.48)			1.50 (0.65−3.29)	0.30
Vasomotor symptoms			0.00 (0.00−0.00)			1.30 (0.00−3.92)	0.07
Psychological symptoms			0.71 (0.43−1.71)			0.57 (0.00−3.82)	0.95
Physical symptoms			1.31 (0.69−1.81)			1.72 (0.84−2.78)	0.45
Sexual health			0.00 (0.00−0.00)			2.50 (0.50−4.50)	0.02
Change (18 mo to baseline)	4	4		4	2		
Overall score			−0.19 (−0.44 to 0.29)			0.69 (−0.44 to 1.77)	0.49
Vasomotor symptoms			0.00 (0.00–0.00)			0.50 (−0.25 to 4.50)	0.69
Psychological symptoms			0.21 (−0.61 to 0.39)			−0.21 (−1.29 to 1.50)	0.69
Physical symptoms			−0.16 (−0.61 to 0.39)			0.88 (−0.28 to 1.38)	0.34
Sexual health			−0.70 (−1.8 to 0.00)			0.83 (0.08 to 3.33)	0.06

*Note:* Variables are given as median and interquartile interval. *p* values were calculated using the Mann-Whitney *U* test. Numbers of patients differ from those in Table 2 because for some patients, data on side effect questionnaires were missing (for the standard of care and direct start of treatment at baseline, n= 5 and n=4; at month 6, n=6; and at month 18, n=7 and n=6, respectively).

Abbreviations: A, available; BD-II, beck depression inventory; MENQOL, Menopause Specific Quality of Life; NA, not available.

### Adverse events

Adverse events are displayed in Table 5. The total number of adverse events (0−18 mo) was significantly higher in the direct-start group compared with the standard-of-care group (38 vs. 9, respectively, *p*<0.001). The majority of adverse events were menopausal in nature and, according to the treating physician, very likely related to treatment, such as mood swings, concentration loss, insomnia, hair loss, and sexual complaints, and, to a lesser extent, muscle and joint pains.

**TABLE 5:** Incidence of adverse events and serious adverse events according to treatment group

	Patients with adverse event, n (%)		
	Standard of care (N=8)	Direct start (N=6)	*p*
Adverse events			
Any	8 (100)	6 (100)	—
Total number of adverse events	9	38	—
Adverse events per patient	1.0 (1.0−1.8)	6.0 (4.5−8.0)	<0.001
Leading to withdrawal	0	1 (16.7)	0.23
Viral infection	2 (25.0)	2 (33.3)	0.73
Heart burn	1 (12.5)	2 (33.3)	0.35
Liver enzyme elevation	2 (25.0)	2 (33.3)	0.73
Albuminuria	1 (12.5)	0	0.37
Bacterial infection	2 (25.0)	1 (16.7)	0.71
Headache	0	1 (16.7)	0.23
Menopausal symptoms summarized	0	5 (83.4)	0.001
Mood swings	0	3 (50.0)	0.02
Concentration loss	0	4 (66.7)	0.006
Elevated creatinine	1 (12.5)	0	0.37
Increased abdominal girth	0	1 (16.7)	0.23
Insomnia	0	2 (33.3)	0.08
Rash	0	1 (16.7)	0.23
Allergic reaction	0	1 (16.7)	0.23
Hypertension	0	2 (33.3)	0.08
Abdominal pain	0	2 (33.3)	0.08
Obstipation	0	1 (16.7)	0.23
Hair loss	0	1 (16.7)	0.23
Sexual complaints	0	2 (33.3)	0.08
Nausea	0	1 (16.7)	0.23
Injection site edema	—	1 (16.7)	—
Muscle pain	0	1 (16.7)	0.23
Joint pain	0	1 (16.7)	0.23
Serious adverse events			
Any	2 (25.0)	0	0.19
Leading to withdrawal	0	—	—
Liver cyst infection	1	—	—
Kidney cyst infection	1	—	—

*Note:* Variables are given as numbers and percentages. *p* values were calculated using the χ^2^ test.

### Liver volumes

An exploratory efficacy outcome of the study was the difference in liver volume between the 2 groups at the various time points (Figure 3B and Table 2). In the direct-start group, the liver volume at baseline was 2.90 L/m (2.49−3.93) and increased to 3.33 L/m (2.52−4.18) after 18 months of treatment. In the standard-of-care group, the liver volume increased from 3.87 L/m (3.18−4.46) at baseline to 4.51 L/m (3.24–5.21) at 18 months. There was no significant difference between the 2 groups at any time point (*p*=0.23 at baseline, *p*=0.18 at 6 mo, and *p*=0.34 at 18 mo).

### Bone density

No significant difference was seen at any time point in the study in bone density, nor in the change of bone density measured with a dual-energy x-ray absorptiometry scan in the direct-start compared with the standard-of-care group (Supplemental Table S2, https://links.lww.com/HC9/C504).

### Kidney volume in participants with ADPKD

Over the entire study period, kidney volume and kidney growth rates in participants with ADPKD were comparable (Supplemental Table S3, https://links.lww.com/HC9/C504). No significant differences were found.

### Other outcomes

All participating women had anti-Müllerian hormone concentrations at baseline and during follow-up compatible with being premenopausal. A significant difference between the 2 groups was noted at baseline for LH, estradiol, and progesterone (Table 1 and Supplemental Table S5, https://links.lww.com/HC9/C504). This was caused by an accidental flaw in the study protocol that required leuprorelin to be started during the luteal phase of the menstrual cycle. In these women, the baseline visit, when blood was drawn for the assessment of hormones, was therefore always in this specific phase of the menstrual cycle, whereas in women in the control group, the baseline visit with blood draw was planned independently of the menstrual cycle. More importantly, Supplemental Table S5, https://links.lww.com/HC9/C504, also shows that estradiol and progesterone decreased sharply during treatment with leuprorelin, in accordance with the intended mechanism of action of this drug. Such an effect was not seen in the control group.

The values for liver function tests, as well as body mass index and upper arm circumference, are provided in Table 1 and in Supplemental Tables S6 and S7, https://links.lww.com/HC9/C504. At baseline and during the study, there were no significant differences in these outcome measures between the 2 treatment groups.

Of note, 2 of the participants in the standard-of-care group received somatostatin analogs. When these 2 patients were excluded from the data set, results were essentially similar and remained nonsignificant.

## DISCUSSION

Patients with PLD can suffer from severe and progressive enlargement of the liver, with associated physical and psychosocial complaints. The treatment options for this disease are limited. In this prospective study, premenopausal women with severe PLD were treated with the GnRH agonist leuprorelin for a maximum of 18 months. The results of this interim analysis showed that the average TLV still increased despite treatment, with 3 out of 6 participants who were still treated at month 18 experiencing an increase in liver volume. In line, leuprorelin did not beneficially influence liver volume–related symptoms as measured with the PLD-Q and SF-36 questionnaires, whereas with this drug, significantly more and severe menopausal side effects and adverse events were reported compared with the standard-of-care group. These findings led to the discontinuation of this study.

The literature on the effects of female sex hormones on liver volume in women with PLD is scarce. A prospective observational study by Sherstha et al^20^ found that estrogen supplementation in 11 postmenopausal women resulted in an increase in liver volume in patients with PLD, compared with 8 controls, where a decrease in liver volume was seen.^20^ Moreover, a cross-sectional study by van Aerts et al^30^ showed that a history of exposure to estrogen-containing oral contraceptives was associated with an increased liver volume in premenopausal women with PLD. These authors found a 15.5% higher hTLV per 10 years of exposure compared with unexposed women with PLD. The number of pregnancies had no influence on the severity of PLD despite the known increase in endogenous estrogen levels during pregnancy.^31^ On the other hand, the larger study of Bazojoo et al^32^ described an increase in liver volume and cyst volume growth rates in 16 females with ADPKD after pregnancy. Regardless of the influence of pregnancy, after menopause, a decrease in liver volume is generally seen in women with PLD.^7^,^21^ Taken together, these studies indicate that there may be a deleterious pathophysiological role for estrogens in the growth of liver volume in premenopausal patients with PLD. This provided the rationale to test the hypothesis that inducing premature menopause through suppression of estrogen and progesterone production could be beneficial in such women. GnRH analogs, such as leuprorelin, are known to downregulate pituitary-ovarian function, leading to cessation of the production of estrogens as well as progesterone and other female hormones.^33^,^34^ Selective estrogen receptor modulators, such as tamoxifen and raloxifene, might be an alternative treatment option to suppress estrogen activity. However, these drugs can act dependent on the surrounding tissue as an agonist or antagonist on α and β estrogen receptors.^35^,^36^ Because of the more predictable suppression of estrogen activity, leuprorelin was used in this study and not a selective estrogen receptor modulator. In general, leuprorelin is reasonably tolerated and used as treatment of, for instance, patients with endometriosis or estrogen receptor–positive mammary carcinoma.^33^,^37^

In accordance with our hypothesis on liver growth, we found a numerically smaller increase in liver volume after treatment with the GnRH agonist leuprorelin. However, the difference between the 2 groups was not statistically significant. This may be due in part to the smaller number of participants included in this interim analysis than intended overall, resulting in limited statistical power. Therefore, an inhibiting effect on liver growth cannot be excluded; however, it should be noted that average liver volume continued to increase in the treated group, with 3 out of 6 participants even showing an increase in liver volume ranging from 3% to 7% per year on treatment. These data indicate that the role of estrogens in the pathophysiology of PLD may need to be re-evaluated, as they do not appear to be the sole driver of cyst growth.

Any potential inhibiting effect of leuprorelin on liver growth must be assessed in the light of possible beneficial effects on liver volume–related complaints and possible negative effects by inducing premature menopause. Although menopausal side effects were expected, they were experienced by participants as more severe than we and women participating in the preparatory focus groups had foreseen. We observed no trend toward fewer intrabdominal volume–related complaints or toward better general quality of life as measured with the SF-36. In contrast, there was a significant negative impact on menopause-related vasomotoric symptoms and sexual health, as measured with the MENQOL questionnaire, and on adverse events. This led 1 woman to stop treatment prematurely, whereas only 2 out of 14 participating women chose to continue leuprorelin treatment in the framework of clinical care after the Data Safety Monitoring Board advised to stop the study.

The major limitation of our study is, as stated before, the limited power due to the small sample size, as the study was terminated prematurely. Furthermore, the 18-month follow-up may have been too short to measure an improvement in quality of life due to treatment. The strengths of our study are that it is the first interventional study to test the hypothesis that estrogens play a role in the treatment of PLD in a randomized setting. The study design was developed after consultation with focus groups consisting of patients with PLD and was carried out by a multidisciplinary team including hepatologists, nephrologists, and gynecologists. Furthermore, the study design was adapted to the mechanism of leuprorelin, which overstimulates the hypothalamus to produce GnRH, leading to desensitization that occurs only after a couple of weeks. To exclude this increase in GnRH as a short-term effect, the change in liver volume was assessed not only from baseline to month 18 but also separately from baseline to month 6, and from month 6 to month 18 after the start of treatment.

In conclusion, we did not find an inhibiting effect of the GnRH agonist leuprorelin on liver growth in premenopausal women with large polycystic livers. The overall treatment efficacy in this study was limited, with the average liver volume still increasing despite treatment. Moreover, we noticed a substantial treatment burden, as indicated by menopausal complaints and other adverse events. These findings in combination led to early study discontinuation.

## Supplementary Material

## Data Availability

Deidentified individual participant data collected during the trial will be made available upon request to researchers who provide a methodologically sound proposal and whose use of the data has been approved by the AGAINST-PLD Steering Committee. Study design and protocol development: S.E. Aapkes, J.P.H. Drenth, and R.T. Gansevoort. Gynecological aspects of study design: M. van den Berg and A.E.P. Cantineau. Imaging aspects of study design: A.L. Messchendorp, R.J. de Haas, and M.D.A. van Gastel. Statistical aspects of the study: P. Vart. Local investigators participating in the design and conduct of the trial: R. Duijzer, P. Geertsema, H. Blokzijl, S.E. Aapkes, and A.L. Messchendorp. Writing of the manuscript G.G. Wijbenga, P. Geertsema, A.L. Messchendorp, and R.T. Gansevoor. All authors read and approved the final manuscript. This study is supported by a grant from the Dutch Organisation for knowledge and innovation in health, healthcare, and well-being (ZonMw grant 10140261910001). Study medication was provided via an unrestricted grant from AbbVie (Hoofddorp, the Netherlands). Neither organization had any role in the conception of this study, nor in the writing of the study protocol and this manuscript.

## References

[1] Van Aerts RMM Van De Laarschot LFM Banales JM Drenth JPH . Clinical management of polycystic liver disease. J Hepatol. 2018;68:827–837. 10.1016/j.jhep.2017.11.024 29175241

[2] Gevers TJ Drenth JP . Diagnosis and management of polycystic liver disease. Nat Rev Gastroenterol Hepatol. 2013;10:101–108.5. 10.1038/nrgastro.2012.254 23296249

[3] Hoevenaren IA Wester R Schrier RW McFann K Doctor RB Drenth JPH . Polycystic liver: Clinical characteristics of patients with isolated polycystic liver disease compared with patients with polycystic liver and autosomal dominant polycystic kidney disease. Liver Int. 2008;28:264–270.6. 10.1111/j.1478-3231.2007.01595.x 17927714

[4] Cornec-Le Gall E Alam A Perrone RD . Autosomal dominant poly-cystic kidney disease. Lancet. 2019;393:919–935.7. 10.1016/S0140-6736(18)32782-X 30819518

[5] Torres VE Harris PC Pirson Y . Autosomal dominant polycystic kidney disease. Lancet. 2007;369:1287–1301. 10.1016/S0140-6736(07)60601-1 17434405

[6] Barten TRM Bernts LHP Drenth JPH Gevers TJG . New insights into targeting hepatic cystogenesis in autosomal dominant polycystic liver and kidney disease. Expert Opin Ther Targets. 2020;24:589–599. 10.1080/14728222.2020.1751818 32250187

[7] Aapkes SE Bernts LHP Barten TRM van den Berg M Gansevoort RT Drenth JPH . Estrogens in polycystic liver disease: A target for future therapies? Liver Int. 2021;41:2009–2019. 10.1111/liv.14986 PMC8456902 34153174

[8] van Aerts RMM Kievit W D’Agnolo HMA Blijdorp CJ Casteleijn NF Dekker SEI . Lanreotide reduces liver growth in patients with autosomal dominant polycystic liver and kidney disease. Gastroenterology. 2019;157:481–491. 10.1053/j.gastro.2019.04.018 31022403

[9] Cnossen WR Drenth JPH . Polycystic liver disease: An overview of pathogenesis, clinical manifestations and management. Orphanet J Rare Dis. 2014;9:69–82. 10.1186/1750-1172-9-69 PMC4030533 24886261

[10] Perugorria MJ Masyuk TV Marin JJ Marzioni M Bujanda L LaRusso NF . Polycystic liver diseases: Advanced insights into the molecular mechanisms. Nat Rev Gastroenterol Hepatol. 2014;11:750–761. 10.1038/nrgastro.2014.155 PMC4526263 25266109

[11] Wijnands TFM Neijenhuis MK Kievit W Nevens F Hogan MC Torres VE . Evaluating health-related quality of life in patients with polycystic liver disease and determining the impact of symptoms and liver volume. Liver Int. 2014;34:1578–1583. 10.1111/liv.12430 24313956

[12] Neijenhuis MK Kievit W Verheesen SMH D’Agnolo HM Gevers TJG Drenth JPH . Impact of liver volume on polycystic liver disease-related symptoms and quality of life. United Eur Gastroenterol J. 2018;6:81–88. 10.1177/2050640617705577 PMC5802666 29435317

[13] European Association for the Study of the Liver . EASL clinical practice guidelines on management of cystic liver diseases. J Hepatol. 2022;77:1083–1108. 10.1016/j.jhep.2022.06.002 35728731

[14] Lanreotide . Farmacotherapeutisch Kompas. Accessed September 19, 2025. www.farmacotherapeutischkompas.nl/bladeren/preparaatteksten/l/lanreotide#bijwerkingen

[15] Van Keimpema L De Koning DB Van Hoek B Van Den Berg AP Van Oijen MGH De Man RA . Patients with isolated polycystic liver disease referred to liver centres: Clinical characterization of 137 cases. Liver Int. 2011;31:92–98. 10.1111/j.1478-3231.2010.02247.x 20408955

[16] Van Keimpema L Nevens F Adam R Porte RJ Fikatas P Becker T . Excellent survival after liver transplantation for isolated polycystic liver disease: An European liver transplant registry study. Transpl Int. 2011;24:1239–1245. 10.1111/j.1432-2277.2011.01360.x 21955068

[17] van Aerts RMM Kievit W de Jong ME Ahn C Bañales JM Reiterová J . Severity in polycystic liver disease is associated with aetiology and female gender: Results of the International PLD Registry. Liver Int. 2019;39:575–582. 10.1111/liv.13965 30225933

[18] Alvaro D Mancino MG Onori P Franchitto A Alpini G Francis H . Estrogens and the pathophysiology of the biliary tree. World J Gastroenterol. 2006;12:3537–3545. 10.3748/wjg.v12.i22.3537 PMC4087569 16773710

[19] Harris RA Gray DW Britton BJ Toogood GJ Morris PJ . Hepatic cystic disease in an adult polycystic kidney disease transplant population. Aust NZ J Surg. 1996;66:166–168. 10.1111/j.1445-2197.1996.tb01148.x 8639135

[20] Sherstha R McKinley C Russ P Scherzinger A Bronner T Showalter R . Postmenopausal estrogen therapy selectively stimulates hepatic enlargement in women with autosomal dominant polycystic kidney disease. Hepatology. 1997;26:1282–1286. 10.1002/hep.510260528 9362373

[21] Chebib FT Jung Y Heyer CM Irazabal MV Hogan MC Harris PC . Effect of genotype on the severity and volume progression of polycystic liver disease in autosomal dominant polycystic kidney disease. Nephrol Dial Transplant. 2016;31:952–960. 10.1093/ndt/gfw008 PMC4876970 26932689

[22] Alvaro D Alpini G Onori P Franchitto A Glaser SS Le Sage G . Alfa and beta estrogen receptors and the biliary tree. Mol Cell Endocrinol. 2002;193:105–108. 10.1016/s0303-7207(02)00103-x 12161009

[23] Alvaro D Onori P Alpini G Franchitto A Jeferson DM Torrice A . Morphological and functional features of hepatic cyst epithelium in autosomal dominant polycystic kidney disease. Am J Pathol. 2008;172:321–332. 10.2353/ajpath.2008.070293 PMC2312356 18202196

[24] Alvaro D Onori P Torrice A Cardinale V Barbaro B Alpini G . Estrogens and Igf1 promote the proliferation of hepatic cyst epithelium in autosomal dominant polycystic kidney disease (ADPKD). Hepatology. 2005;42:202A.

[25] Aapkes SE Bernts LHP van den Berg AP van den Berg M Blokzijl H Cantineau AEP . Protocol for a randomized controlled multicenter trial assessing the efficacy of leuprorelin for severe polycystic liver disease: The AGAINST-PLD study. BMC Gastroenterol. 2022;22:82 10.1186/s12876-022-02142-y PMC8876117 35216547

[26] D’Agnolo HMA Kievit W Andrade RJ Karlsen TH Wedemeyer H Drenth JPH . Creating an effective clinical registry for rare diseases. United Eur Gastroenterol J.. 2016;4:333–338. 10.1177/2050640615618042 PMC4924439 27403298

[27] van Gastel MDA Edwards ME Torres VE Erickson BJ Gansevoort RT Kline TL . Automatic measurement of kidney and liver volumes from MR images of patients affected by autosomal dominant polycystic kidney disease. J Am Soc Nephrol. 2019;30:1514–22. 10.1681/ASN.2018090902 PMC6683702 31270136

[28] Gevers TJG Inthout J Caroli A Ruggenenti P Hogan MC Torres VE . Young women with polycystic liver disease respond best to somatostatin analogues: A pooled analysis of individual patient data. Gastroenterology. 2013;145:357–365. 10.1053/j.gastro.2013.04.055 23665274

[29] Hogan MC Masyuk TV Page LJ Kubly VJ Bergstralh EJ Li X . Randomized clinical trial of long-acting somatostatin for autosomal dominant polycystic kidney and liver disease. J Am Soc Nephrol. 2010;21:1052–1061. 10.1681/ASN.2009121291 PMC2900957 20431041

[30] van Aerts RMM Bernts LHP Gevers TJG Kievit W Koopmans L Nieboer TE . Estrogen-containing oral contraceptives are associated with polycystic liver disease severity in premenopausal patients. Clin Pharmacol Ther. 2019;160:1338–1345. 10.1002/cpt.1553 PMC6896230 31206615

[31] Toriola AT Vääräsmäki M Lehtinen M Zeleniuch-Jacquotte A Lundin E Rodgers KG . Determinants of maternal sex steroids during the first half of pregnancy. Obstet Gynecol. 2011;118:1029–1036. 10.1097/AOG.0b013e3182342b7f PMC3207141 22015870

[32] Bazojoo V Davoudi V Blumenfeld JD Zhu C Malha L Lo GC . Effects of pregnancy on liver and kidney cyst growth rates in autosomal dominant polycystic kidney disease: A pilot study. J Clin Med. 2025;14:3688 10.3390/jcm14113688 PMC12156408 40507450

[33] Cheung TK Lo KW Lam CW Lau W Lam PK . A crossover study of triptorelin and leuprorelin acetate. Fertil Steril. 2000;74:299–305. 10.1016/s0015-0282(00)00598-7 10927048

[34] Tarlatzis BC Bili H . Safety of GnRH agonists and antagonist. Expert Opin Drug Saf. 2004;3:39–46. 10.1517/14740338.3.1.39 14680460

[35] Pinkerton JV Conner EA . Beyond estrogen: Advances in tissue selective estrogen complexes and selective estrogen receptor modulators. Climacteric. 2019;22:140–147. 10.1080/13697137.2019.1568403 30895900

[36] Ellis AJ Hendrick VM Williams R Komm BS . Selective estrogen receptor modulators in clinical practice: A safety overview. Expert Opin Drug Saf. 2015;14:921–934. 10.1517/14740338.2015.1014799 25936229

[37] Periti P Mazzei T Mini E . Clinical pharmacokinetics of depot leuprorelin. Clin Pharmacokinet. 2002;41:485–504. 10.2165/00003088-200241070-00003 12083977

