# Supplementary material for: Effects of the GnRH agonist leuprorelin on liver volume in premenopausal women with severe polycystic liver disease
Source: Hepatol Commun. 2026 Oct 19;10(11):e1067. doi: 10.1097/HC9.0000000000001067 (PMC13682819; doi:10.1097/HC9.0000000000001067)
Supplement: Supplementary file 1 [file hc9-10-e1067-s001.docx]

|  | **Leuprorelin** | | | | | | |  |
| --- | --- | --- | --- | --- | --- | --- | --- | --- |
|  | **Standard of care** | | |  | **Direct start** | | |  |
|  | A |  |  |  | A |  |  | P-val. |
| **Primary outcome** |  |  |  |  |  |  |  |  |
| Liver growth rate (%/year)^1^ |  |  |  |  |  |  |  |  |
| - First study period (0-6 months) | 8 |  | 9.56±8.26 |  | 6 |  | 1.31±7.65 | 0.052 |
| - Second study period (6-18 months) | 7 |  | 7.45±10.1^#^ |  | 5 |  | 2.10±7.39^#^ | 0.34 |
| - Total study period (0-18 months) | 7 |  | 8.18±7.87 |  | 5 |  | 1.70±5.90 | 0.14 |
| Variables are presented as mean± standard deviation. P-values are calculated using an unpaired t-test. Abbreviations are: A, available; NA, non-available; P-val., P-value. Data of all patients was complete.  ^#^ First versus second study period, p=0.50 (standard of care) and p=0.89 (direct start) | | | | | | | | |

**Supplemental Table 1.** Analysis of liver growth using t-test, as defined a priori.

**Supplemental Table 2.** Bone density as measured with a DEXA-scan according to treatment group.

|  | **Leuproprelin** | | | | | | |  |
| --- | --- | --- | --- | --- | --- | --- | --- | --- |
|  | **Standard of care** | | |  | **Direct start** | | | **P-value** |
| **DEXA-scan T-score** | A | NA |  |  | A | NA |  |  |
| Baseline | 8 | *-* |  |  | 6 | *-* |  |  |
| - Continuous |  |  | -0.5 (-1.4-0.3) |  |  |  | -0.1 (-1.0-0.5) | 0.66 |
| - Categorical, n (%) |  |  |  |  |  |  |  | 0.71 |
| ≥-1.0 (normal) |  |  | 6 (75.0) |  |  |  | 5 (83.3) |  |
| -1.0 to -2.5 (osteopenia) |  |  | 2 (25.0) |  |  |  | 1 (16.7) |  |
| ≤ -2.5 (osteoporosis) |  |  | 0 (0) |  |  |  | 0 (0) |  |
| 18 months | 6 | *1^*^* |  |  | 6 | *-* |  |  |
| - Continuous |  |  | -1.4 (-2.0-0.0) |  |  |  | -1.1 (-1.7-0.5) | 0.70 |
| - Categorical, n (%) |  |  |  |  |  |  |  | 0.55 |
| ≥-1.0 (normal) |  |  | 3 (50.0) |  |  |  | 3 (50.0) |  |
| -1.0 to -2.5 (osteopenia) |  |  | 3 (50.0) |  |  |  | 2 (33.3) |  |
| ≤ -2.5 (osteoporosis) |  |  | 0 (0) |  |  |  | 1 (16.7) |  |
| Change (18 months-baseline) | 6 | *1^*^* | -0.3(-0.9- 0.05) |  | 6 | *-* | -1.1 (-1.3--0.2) | 0.24 |
| ^*^DEXA-scan was not performed in 1 patient at 18 months. P-values were calculated using Mann-Whitney U test and Chi-square tests. Abbreviations are: A, available; DEXA; dual-energy X-ray absorptiometry; NA, non-available. | | | | | | | | |

**Supplemental Table 3.** Kidney volume according to treatment group^*^.

|  | **Leuprorelin** | | | | | | | |  |
| --- | --- | --- | --- | --- | --- | --- | --- | --- | --- |
|  | **Standard of care** | | |  | **Direct start** | | | |  |
|  | A |  |  |  | A |  | |  | P-val. |
| **PKD specific outcomes^**^** |  |  |  |  |  |  |  | |  |
| Kidney growth rate (%/year)^1^ |  |  |  |  |  |  |  | |  |
| - First study period (0-6 months) | 7 |  | -2.78 (-18.0- 0.00) |  | 4 |  | -3.08 (-12.0-16.0) | | 0.65 |
| - Second study period (6-18 months) | 6 |  | 5.95^$^ (-2.72-15.8) |  | 4 |  | 2.25^$^ (0.38-14.4) | | 0.76 |
| - Total study period (0-18 months) | 6 |  | 2.49 (-4.90-8.80) |  | 4 |  | 3.17 (-0.63-6.01) | | 0.91 |
| Kidney volume (mL/m) |  |  |  |  |  |  |  | |  |
| - Baseline | 7 |  | 719 (353-1144) |  | 4 |  | 456 (334-568) | | 0.23 |
| - 6 months | 7 |  | 718 (344-1337) |  | 4 |  | 464 (334-544) | | 0.41 |
| - 18 months | 6 |  | 988 (372-1382) |  | 4 |  | 481 (338-602) | | 0.26 |
| Change in eGFR (mL/min/1.73 m^2^ per year) |  |  |  |  |  |  |  | |  |
| - First study period (0-6 months) | 7 |  | -2.0 (-3.5-0.5) |  | 4 |  | -1.0 (-8.3-4.0) | | 0.92 |
| - Second study period (6-18 months) | 6 |  | 1.0 (-0.8-3.5) |  | 4 |  | 2.0 (-0.5-3.8) | | 0.75 |
| - Total study period (0-18 months) | 6 |  | -0.5 (-5.8-1.8) |  | 4 |  | -3.0 (-6.0;1.0) | | 0.99 |

| **PKD specific outcomes^***^** |  |  |  |  |  |  |  |  |
| --- | --- | --- | --- | --- | --- | --- | --- | --- |
| Kidney growth rate (%/year)^1^ |  |  |  |  |  |  |  |  |
| - First study period (0-6 months) | 7 |  | -2.78 (-18.0-0) |  | 4 |  | -3.08(-12.0-16.0) | 0.65 |
| - Second study period (6-18 months) | 6 |  | 5.95^#^ (-2.72-15.8) |  | 3 |  | 2.41^#^ (-0.19-18.4) | 0.71 |
| - Total study period (0-18 months) | 6 |  | 2.49 (-4.90-8.80) |  | 3 |  | 4.65 (1.69-6.46) | 0.91 |
| Kidney volume (mL/m) |  |  |  |  |  |  |  |  |
| - Baseline | 7 |  | 719 (353-1144) |  | 4 |  | 456 (334-568) | 0.23 |
| - 6 months | 7 |  | 719 (344-1337) |  | 4 |  | 464 (334-544) | 0.41 |
| - 18 months | 6 |  | 988 (372-1382) |  | 3 |  | 562 (400-615) | 0.38 |
| Change in eGFR (mL/min/1.73 m^2^ per year) |  |  |  |  |  |  |  |  |
| - First study period (0-6 months) | 7 |  | -2.0 (-3.5-0.5) |  | 4 |  | -1.0 (-8.3-4.0) | 0.92 |
| - Second study period (6-18 months) | 6 |  | 1.0 (-0.8-3.5) |  | 3 |  | 3.0 (-1.0-4.5) | 0.70 |
| - Total study period (0-18 months) | 6 |  | -0.5 (-5.8-1.8) |  | 3 |  | -1.0 (-5.0;3.0) | 0.90 |
| Variables are presented as median (IQI, interquartile interval). P-values are calculated using Mann Whitney U for cross-sectional comparisons. For paired analyses Wilcoxon signed ranks test was used. Abbreviations are: A, available; eGFR, estimated glomerular filtration rate; NA, non-available; number; P-val., P-value; PKD, polycystic kidney disease. Data of all patients was complete.  ^*^Only including subjects with ADPKD  ^**^Intention to treat analysis  ^***^Per protocol analysis  ^$^ First versus second study period, p=0.17 (standard of care) and p=0.59 (direct start)  ^#^ First versus second study period, p=0.17 (standard of care) and p=0.59 (direct start) | | | | | | | | |

**Supplemental Table 4.** Intention to treat analysis of liver volume according to treatment group.

|  | **Leuprorelin** | | | | | | |  |
| --- | --- | --- | --- | --- | --- | --- | --- | --- |
|  | **Standard of care** | | |  | **Direct start** | | |  |
|  | A |  |  |  | A |  |  | P-val. |
| **Primary outcome** |  |  |  |  |  |  |  |  |
| Liver growth rate (%/year) |  |  |  |  |  |  |  |  |
| - First study period (0-6 months) | 8 |  | 11.6  (0.83-16.5) |  | 6 |  | 0.45  (-4.88-6.90) | 0.14 |
| - Second study period (6-18 months) | 7 |  | 10.6^#^  (-0.74-12.6) |  | 6 |  | 6.31^#^  (-4.44-12.8) | 0.63 |
| - Total study period (0-18 months) | 7 |  | 11.0  (0.70-13.2) |  | 6 |  | 5.20  (-4.40-8.68) | 0.30 |
| Linear mixed effect models (%/year (CI)) |  |  |  |  |  |  |  |  |
| - First study period (0-6 months) | 8 |  | 8.08  (1.89 to 15.0) |  | 6 |  | 0.65  (-6.37 to 8.41) | 0.17 |
| - Second study period (6-18 months) | 7 |  | 6.58^†^  (0.80 to 12.6) |  | 6 |  | 5.04^†^  (-1.04 to 11.7) | 0.74 |
| - Total study period (0-18 months) | 8^§^ |  | 7.04  (1.91 to 12.4) |  | 6 |  | 3.42  (-2.11 to 9.26) | 0.38 |
| **Exploratory outcomes** |  |  |  |  |  |  |  |  |
| Liver volume (L/m) |  |  |  |  |  |  |  |  |
| - Baseline | 8 |  | 3.87  (3.18-4.46) |  | 6 |  | 2.90  (2.49-3.93) | 0.23 |
| - 6 months | 8 |  | 4.17  (3.20-4.65) |  | 6 |  | 2.86  (2.53-3.88) | 0.18 |
| - 18 months | 7 |  | 4.51  (3.24-5.21) |  | 6 |  | 3.27  (2.61-3.84) | 0.23 |
| Variables are presented as mean ±SD. P-values are calculated using Mann Whitney U for cross-sectional comparisons. For paired analyses a paired-test was used. Data of all patients was complete.  Abbreviations are: A, available; CI, confidence interval; NA, non-available; P-val., P-value.  ^#^ First versus second study period, p=0.50 (standard of care) and p=0.30 (direct start)  ^†^ First versus second study period, p=0.69 (standard of care) and p=0.30 (direct start)  ^§^ Liver growth rate during the total study period estimated by linear mixed effects modeling included measurements of the first study period from one patient in the standard of care group | | | | | | | | |

**Supplemental Table 5.** Intention to treat analysis of sex hormones according to treatment group.

|  | **Leuprorelin** | | | | | | |  |
| --- | --- | --- | --- | --- | --- | --- | --- | --- |
|  | **Standard of care** | | |  | **Direct start** | | |  |
|  | A |  |  |  | A |  |  | P-val. |
| Estradiol (nmol/L) |  |  |  |  |  |  |  |  |
| - Baseline | 7 |  | 0.28  (0.23-0.32) |  | 4 |  | 0.49  (0.39-0.63) | 0.02 |
| - 6 months | 7 |  | 0.19  (0.16-0.22) |  | 6 |  | 0.03  (0.02-0.19) | 0.05 |
| - 18 months | 3 |  | 0.23  (0.11-NA) |  | 2 |  | 0.08  (0.05-NA) | NA |
| Progesteron (nmol/L) |  |  |  |  |  |  |  |  |
| - Baseline | 7 |  | 8.60  (0.18-26.0) |  | 4 |  | 43.5  (15.4-55.1) | 0.11 |
| - 6 months | 7 |  | 0.53  (0.21-14.5) |  | 6 |  | 0.10  (0.09-0.20) | 0.01 |
| - 18 months | 3 |  | 3.07  (0.51-NA) |  | 2 |  | 0.52  (0.10-NA) | NA |
| LH (U/L) |  |  |  |  |  |  |  |  |
| - Baseline | 7 |  | 6.8  (4.8-8.2) |  | 4 |  | 39.3  (32.1-55.5) | 0.008 |
| - 6 months | 7 |  | 8.7  (4.6-14.4) |  | 6 |  | 1.2  (0.4-5.0) | 0.02 |
| - 18 months | 3 |  | 18.5  (4.4-NA) |  | 2 |  | 2.3  (0.9-NA) | NA |
| AMH (μg/L) |  |  |  |  |  |  |  |  |
| - Baseline | 7 |  | 0.90  (0.37-1.80) |  | 4 |  | 2.00  (0.67-2.76) | 0.26 |
| - 6 months | 7 |  | 0.69  (0.28-1.75) |  | 6 |  | 1.42  (0.18-2.04) | 0.57 |
| - 18 months | 3 |  | 0.57  (0.38-NA) |  | 2 |  | 2.36  (2.21-NA) | NA |
| Variables are presented as median (IQI, interquartile interval). P-values are calculated using a Mann Whitney U test. Abbreviations are: A, available; CI, confidence interval; NA, non-available; P-val., P-value. | | | | | | | | |

**Supplemental Table 6.** Intention to treat analysis of liver test biochemistry and CA19.9 levels according to treatment group.

|  | **Leuprorelin** | | | | | | |  |
| --- | --- | --- | --- | --- | --- | --- | --- | --- |
|  | **Standard of care** | | |  | **Direct start** | | |  |
|  | A |  |  |  | A |  |  | P-val. |
| ASAT (U/L) |  |  |  |  |  |  |  |  |
| - Baseline | 8 |  | 22  (20-29) |  | 6 |  | 32  (21-46) | 0.17 |
| - 6 months | 8 |  | 22  (20-24) |  | 6 |  | 31  (25-36) | 0.06 |
| - 18 months | 7 |  | 23  (19-26) |  | 6 |  | 31  (21-49) | 0.17 |
| ALAT (U/L) |  |  |  |  |  |  |  |  |
| - Baseline | 8 |  | 19  (12-25) |  | 6 |  | 28  (23-59) | 0.07 |
| - 6 months | 8 |  | 18  (12-23) |  | 6 |  | 26  (21-49) | 0.07 |
| - 18 months | 7 |  | 17  (12-21) |  | 6 |  | 27  (20-60) | 0.07 |
| AF (U/L) |  |  |  |  |  |  |  |  |
| - Baseline | 8 |  | 84  (67-108) |  | 6 |  | 77  (55-181) | 0.77 |
| - 6 months | 8 |  | 81  (68-101) |  | 6 |  | 90  (78-161) | 0.44 |
| - 18 months | 7 |  | 76  (70-99) |  | 6 |  | 102  (81-294) | 0.20 |
| GGT (U/L) |  |  |  |  |  |  |  |  |
| - Baseline | 8 |  | 75  (46-119) |  | 6 |  | 75  (37-413) | 0.90 |
| - 6 months | 8 |  | 64  (46-108) |  | 6 |  | 91  (46-269) | 0.37 |
| - 18 months | 7 |  | 88  (47-89) |  | 6 |  | 102  (56-510) | 0.39 |
| Total bilirubin (μmol/L) |  |  |  |  |  |  |  |  |
| - Baseline | 7 |  | 14  (11-23) |  | 5 |  | 15  (9-20) | 0.87 |
| - 6 months | 7 |  | 14  (11-21) |  | 6 |  | 12  (8-16) | 0.22 |
| - 18 months | 6 |  | 14  (9-33) |  | 5 |  | 12  (7-23) | 0.52 |
| CA19.9 (kU/L) |  |  |  |  |  |  |  |  |
| - Baseline | 7 |  | 16  (10-27) |  | 4 |  | 12  (2-33) | 0.64 |
| - 6 months | 7 |  | 11  (8-24) |  | 6 |  | 18  (3-27) | 0.83 |
| - 18 months | 3 |  | 15  (11-NA) |  | 2 |  | 24  (5-NA) | NA |
| Variables are presented as median (IQI, interquartile interval). P-values are calculated using a Mann Whitney U test. Abbreviations are: A, available; CI, confidence interval; NA, non-available; P-val., P-value. | | | | | | | | |

**Supplemental Table 7.** Intention to treat analysis of body mass index (BMI) and upper arm circumference according to treatment group.

|  | **Leuprorelin** | | | | | | |  |
| --- | --- | --- | --- | --- | --- | --- | --- | --- |
|  | **Standard of care** | | |  | **Direct start** | | |  |
|  | A |  |  |  | A |  |  | P-val. |
| Body mass index (kg/m^2^) |  |  |  |  |  |  |  |  |
| - Baseline | 8 |  | 24.5  (22.9-28.0) |  | 6 |  | 23.7  (20.8-24.6) | 0.37 |
| - 6 months | 8 |  | 24.5  (23.0-27.7) |  | 6 |  | 23.6  (21.2-24.6) | 0.37 |
| - 18 months | 7 |  | 25.2  (24.1-30.5) |  | 6 |  | 23.6  (21.2-24.6) | 0.25 |
| Upper arm circumference (cm) |  |  |  |  |  |  |  |  |
| - Baseline | 8 |  | 25.0  (22.8-27.8) |  | 6 |  | 25.0  (23.3-26.5) | 0.70 |
| - 6 months | 8 |  | 25.0  (24.0-26.8) |  | 6 |  | 24.5  (22.8-27.0) | 0.70 |
| - 18 months | 7 |  | 26.0  (24.0-27.0) |  | 6 |  | 22.5  (20.5-26.0) | 0.20 |
| Variables are presented as median (IQI, interquartile interval). P-values are calculated using a Mann Whitney U test. Abbreviations are: A, available; CI, confidence interval; NA, non-available; P-val., P-value. | | | | | | | | |

**Supplemental Figure 1.** Representative MR image without (left panel) and with volumetry (right panel) showing presence of numerous liver cysts and a marked increase in liver volume. Liver volume (LV) in this female patient of 45 years, with a weight of 75 kg and a height of 1.80 m, was 8137 mL. The volume of the right kidney was 1067 mL and of the left kidney 1288 mL (total kidney volume (TKV) being 2355 mL, height adjusted TKV (htTKV) being 1308 mL/m, which represents Mayo Imaging Class 1D). The volume of a normal, nonpolycystic liver and kidney is around 1500 mL and 150 mL, respectively.
